# Pathogenic Process-Associated Transcriptome Analysis of *Stemphylium lycopersici* from Tomato

**DOI:** 10.1155/2022/4522132

**Published:** 2022-05-20

**Authors:** Dezhen Zhang, Wenjuan Chi, Cuicui Wang, Huijie Dai, Jintang Li, Chunlei Li, Fajun Li

**Affiliations:** Shandong Facility Horticulture Bioengineering Research Center, Weifang University of Science and Technology, Weifang 262700, China

## Abstract

Tomato (*Solanum lycopersicum*) gray leaf spot disease is a predominant foliar disease of tomato in China that is caused mainly by the necrotrophic fungal pathogen *Stemphylium lycopersici*. Little is known regarding the pathogenic mechanisms of this broad-host-range pathogen. In this study, a comparative transcriptomic analysis was performed and more genetic information on the pathogenicity determinants of *S*. *lycopersici* during the infection process in tomato were obtained. Through an RNA sequencing (RNA-seq) analysis, 1,642 and 1,875 genes upregulated during the early infection and necrotrophic phases, respectively, were identified and significantly enriched in 44 and 24 pathways, respectively. The induction of genes associated with pectin degradation, adhesion, and colonization was notable during the early infection phase, whereas during the necrotrophic phase, some structural molecule activity-related genes were prominently induced. Additionally, some genes involved in signal regulation or encoding hemicellulose- and cellulose-degrading enzymes and extracellular proteases were commonly upregulated during pathogenesis. Overall, we present some putative key genes and processes that may be crucial for *S*. *lycopersici* pathogenesis. The abilities to adhere and colonize a host surface, effectively damage host cell walls, regulate signal transduction to manage infection, and survive in a hostile plant environment are proposed as important factors for the pathogenesis of *S*. *lycopersici* in tomato. The functional characterization of these genes provides an invaluable resource for analyses of this important pathosystem between *S*. *lycopersici* and tomato, and it may facilitate the generation of control strategies against this devastating disease.

## 1. Introduction

Tomato (*Solanum lycopersicum*) gray leaf spot disease is considered a major damaging, even devastating, disease of cultivated tomatoes and has threatened tomato-growing areas worldwide [1]. As a foliar disease, it usually occurs and develops under warm air temperature and high-humidity conditions [2], and the leaf damage reduces fruit quality and yield [3, 4].

*Stemphylium lycopersici* and *Stemphylium solani* are both causative agents of gray leaf spot disease on tomato [5, 6], and they have been widely reported in many countries. As two closely related species, *S*. *lycopersici* and *S*. *solani* often cause almost indistinguishable symptoms. More importantly, as necrotrophic parasitic fungi, they have wide host ranges that include important vegetable crops, such as tomato [7, 8], pepper [9], and eggplant [3]. In recent years, several new hosts, such as lettuce [10], physali [11], asparagus [12], and watermelon [13], have been discovered and reported in certain areas. Therefore, the pathogenic mechanisms of the two species of *Stemphylium* against plants need to be well studied to effectively control the diseases that they cause.

For the past few years, research on the fungal pathogens *S*. *lycopersici* and *S*. *solani* has mainly focused on biological characteristics [14], histology [15], genetic and virulence variability levels [16], and phytotoxins [17]. Compared with other filamentous fungal pathogens, studies on the molecular mechanisms underlying the interactions of *S*. *lycopersici* and *S*. *solani* with their host, as well as on cloning of virulence-associated genes, are still limited. The whole-genome sequences of *S*. *lycopersici* strains CIDEFI-216, CIDEFI-212, and CIDEFI-213 have been reported and deposited in GenBank, increasing the possibilities and convenient resources for the study of molecular mechanisms of plant pathogenic diseases [18, 19]. Zeng et al. [20] have predicted *S*. *lycopersici*-secreted proteins, but the correlation between the virulence and secreted proteins has not been validated. Furthermore, little is known about the pathogenic molecular mechanisms, such as those involved in the infection process, disrupting host cells and reproduction in the host.

Pathogen infection and the colonization of host plants are complicated dynamic processes. High-throughput transcriptomic sequencing is a suitable approach for investigating the complex interplay between gene expression and regulation under specific conditions or phases [21] that will facilitate systematically revealing the complicated regulatory mechanisms behind pathogenesis and the relationships between the pathogens *S*. *lycopersici* and *S*. *solani* and the host.

In this study, *S*. *lycopersici* strain SL1216 isolated from diseased leaves of tomato was investigated. Using an RNA-seq technique, gene expression during the infection and colonization phases of *S*. *lycopersici* was determined and compared. These research results provide valuable reference information to help reveal the molecular mechanisms of *S*. *lycopersici* pathogenicity, as well as interactions between tomato and *S*. *lycopersici*.

## 2. Materials and Methods

### 2.1. Biological Material and Inoculation Assays

We isolated *S*. *lycopersici* strain SL1216 from infected tomato leaves obtained from a commercial tomato greenhouse in Shouguang, Shandong Province, China, and it was highly virulent in tomato. Tomato plants (*S*. *lycopersicum* “Jinpeng No. 1”) grown individually in plastic pots (10 cm diameter × 8 cm height) were placed in a growth chamber set at 25°C under a 16 h light : 8 h dark photoperiod.

The *S*. *lycopersici* spores were harvested from fungal growth on V8 juice agar plates [22] at 25°C for 14 d under a 12 h light : 12 h dark photoperiod and then adjusted to 1 × 10^5^ spores/mL with sterile ddH_2_O. The spore suspension was dropped on isolated tomato leaves, and the leaves were cultured in an artificial climate incubator at 25°C with 100% relative humidity under a 12 h light : 12 h dark photoperiod. The symptoms of infected leaves were observed at 0, 24, 48, 72, and 96 h postinoculation (hpi). Images were taken of the typical symptoms of infected tomato leaves at different time points. The spore suspension was inoculated into a PD medium and incubated at 200 rpm, 25°C for 36 h, to obtain newly germinated filaments as the control sample.

### 2.2. Mycelial Collection and RNA Extraction

The spores and mycelia were collected at 36 hpi and 84 hpi together with infected tomato leaves, and total RNA was extracted from samples taken at each time point using the TaKaRa MiniBEST universal RNA extraction kit following the manufacturer's instructions. Newly germinating spores cultured for 36 h were collected and processed as described in Section 2.1 for the control sample. The test was conducted using triplicate biological replicates for every treatment (Con, 36 hpi, and 84 hpi). Nine samples were therefore collected. Total RNA was quantified and assessed for purity to meet the sequencing requirements.

### 2.3. RNA Sequencing and Read Assembly

Equal quantities of high-quality RNA for each sample were used to construct the sequencing libraries with a VAHTS mRNA-seq v2 Library Prep Kit (Illumina, Inc., San Diego, CA). Each transcriptome was sequenced on an Illumina HiSeq X Ten (PE 150) instrument at Novogene Corporation, Beijing, China, in accordance with the manufacturer's standard protocol. Raw reads were processed using Trimmomatic [23]. The reads containing ploy-N and the low-quality reads were removed to obtain the clean reads. The clean reads were assembled into expressed sequence tag clusters (contigs) and de novo assembled into the transcript by using Trinity [24] (version: 2.4) in the paired-end method. The TGI clustering tools [25] were used to cluster assembly sequences and remove redundancies to produce a unigene dataset. The unigenes were mapped to the *S*. *lycopersici* genome to further exclude contaminating sequences. The resulting transcript dataset was taken as a *S*. *lycopersici* reference transcriptome for subsequent analyses.

### 2.4. Transcriptome Annotation and Differential Expression Analysis

The assembled unigenes were annotated on the basis of the top hits of a BLASTX search against various protein databases. The Blast2GO [26] program was employed to obtain a functional annotation of all the unigenes using the Gene Ontology (GO) database. The clean reads were aligned to the assembled unigenes by SOAP2 [27]. The expression levels based on the number of reads uniquely mapped to a unigene were normalized using the fragments per kb per million reads method. Differentially expressed genes (DEGs) were analyzed using the DESeq2 package [28] based on the negative binomial distribution test. The thresholds of DEGs were set as false discovery rate (FDR) ≤ 0.05 and |log2 fold change| ≥ 1 in this paper.

The GO enrichment analysis of DEGs was implemented with a perl module (GO::TermFinder) [29]. The statistical enrichment of the DEGs among the Kyoto Encyclopedia of Genes and Genomes (KEGG) pathways was tested using R functions (phyper and qvalue). For GO terms and KEGG pathways, corrected *p* values less than 0.05 were considered to be significantly enriched among the differentially expressed unigenes.

### 2.5. RNA-seq Validation by Quantitative Real-Time PCR (qRT-PCR)

To validate the expression profiles obtained by RNA-seq, the expression levels of eight randomly selected DEGs were analyzed by performing qRT-PCR. Specific primer pairs were designed for the eight DEG sequences in https://sg.idtdna.com/Primerquest/Home/Index. The actin gene was used as the internal control gene to obtain more accurate quantitative results (Table S1). The synthesis of first-strand cDNA from each sample was performed using EasyScript All-in-One First-Strand cDNA Synthesis SuperMix for qPCR (One-Step gDNA Removal, TransGen, China) in accordance with the manufacturer's instructions. qRT-PCR was performed using SYBR® Premix Ex Taq™ II (Tli RNaseH Plus, TaKaRa) on a Mastercycler Ep Realplex system (Eppendorf, Hamburg, Germany). The amplifications were performed in 20 *μ*L reactions containing 10 *μ*L of 2x TransStart® Top Green qPCR SuperMix (TransGen), 0.4 *μ*L of each primer (10 *μ*M), 2 *μ*L diluted cDNA (1–100 ng), and 7.2 *μ*L of nuclease-free water. The samples were incubated at 94°C for 30 s as an initial denaturation, followed by 40 amplification cycles at 94°C for 5 s, 60°C for 15 s, and 72°C for 20 s. The relative expression quantification of each DEG was determined using the 2^−*ΔΔ*Ct^ method [30]. These qRT-PCR assays were performed with three biological replicates.

## 3. Results

### 3.1. Time Course of *S*. *lycopersici* Infection on Tomato Leaves

**Figure 1:**
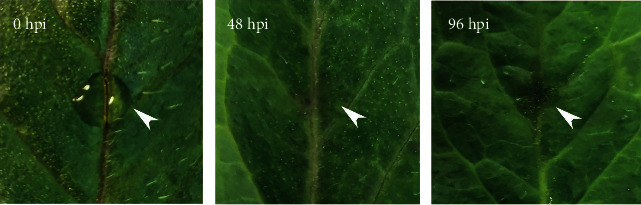
Symptom development on tomato leaves inoculated with a spore suspension of *S*. *lycopersici*. The spore suspension was inoculated on tomato leaves (0 hpi), brown spots began to appear at 48 hpi, and significant local leaf cellular necrosis was observed by 96 hpi.

Isolated tomato leaves were inoculated with a spore suspension, and the symptom development process was observed (Figure 1). At 48 hpi, brown spots began to appear at the leaf inoculation sites. However, we observed significant local leaf cellular necrosis by 96 hpi. Because pathogenesis-related gene expression tended to precede symptom onset, we considered 36 hpi with *S*. *lycopersici* as the early infection phase, whereas 84 hpi was considered the necrotrophic phase. Samples were taken at these two time points for transcriptome sequencing to investigate the dynamics of pathogenesis-related genes during *S*. *lycopersici* infection of tomato.

### 3.2. De Novo Assembly of the *S*. *lycopersici* Transcriptome

Using RNA-seq technology, nine transcriptomic datasets were generated from control (Con), 36 hpi, and 84 hpi samples to better understand the pathogenicity of *S*. *lycopersici*. We obtained 66.15 Gb raw bases and 60.08 Gb clean bases. An overview of the transcriptome assembly statistics is shown in Table 1. After removing low-quality and adapter sequences, a total of 137.23 M, 141.27 M, and 140.78 M clean reads were obtained for Con, 36 hpi, and 84 hpi samples, respectively. The average Q30 values were greater than 92.50%, and the average GC values of the Con, 36 hpi, and 84 hpi samples were 54.97%, 55.33%, and 55.28%, respectively.

### 3.3. DEGs of *S*. *lycopersici*

**Figure 2:**
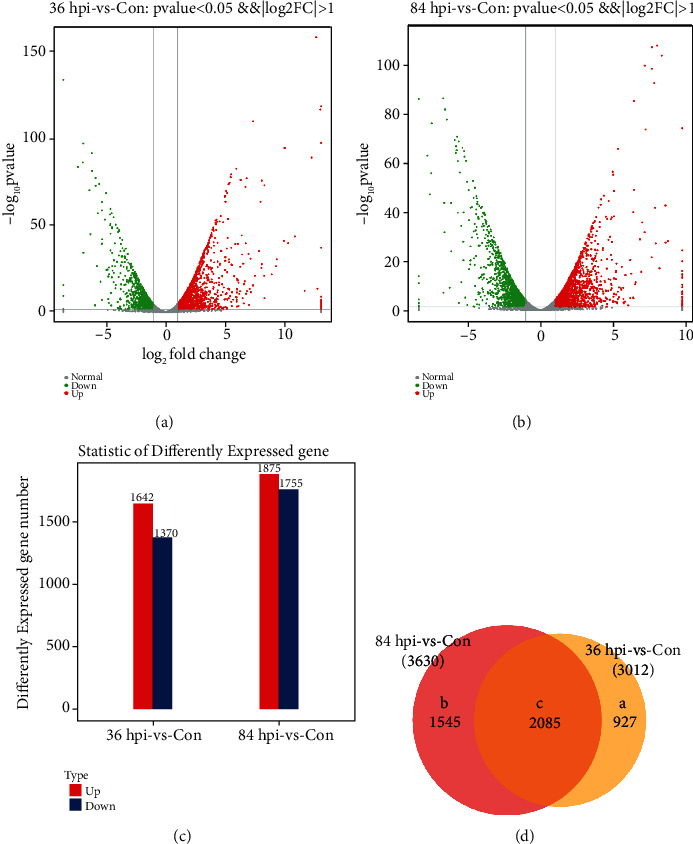
Differentially expressed genes (DEGs) of *S*. *lycopersici*. (a) Volcano plot of the global comparison of transcript profiles between Con and 36 hpi. Gray dots indicate genes of no significance. Red dots indicate upregulated genes meeting the thresholds of log2 fold change > 1 and padj < 0.05. Green dots indicate downregulated genes meeting the thresholds of log2 fold change < −1 and padj < 0.05. (b) Volcano plot of the global comparison of transcript profiles between Con and 84 hpi. (c) Statistical histogram of the DEGs. Red and blue columns represent the up- and downregulated genes, respectively. (d) Venn plot of common and unique DEGs between the different comparison groups. Region a represents the number of DEGs only in 36 hpi-vs-Con. Region b represents the number of DEGs only in 84 hpi-vs-Con. Region c represents the number of DEGs present in both comparison groups.

The global distribution of DEGs in 36 hpi-vs-Con and 84 hpi-vs-Con was determined using a volcano plot (Figures 2(a) and 2(b)). A total of 3,012 and 3,630 DEGs were identified at 36 hpi and 84 hpi, respectively, compared with the Con (*p* value < 0.05 and |log2 fold change| > 1). Overall, the majority of the DEGs (1,642, 54.52%) in 36 hpi-vs-Con were upregulated, whereas 1,875 (51.65%) DEGs were upregulated in 84 hpi-vs-Con (Figure 2(c)). Among the DEGs, 2,850 were common to 36 hpi-vs-Con and 84 hpi-vs-Con, whereas 927 and 1,545 DEGs were unique in 36 hpi-vs-Con and 84 hpi-vs-Con, respectively (Figure 2(d)). The presence of unique DEGs in different comparison groups suggested that different physiological activities were performed in *S*. *lycopersici* during the various infection stages.

### 3.4. *S*. *lycopersici* Transcriptome Annotation

#### 3.4.1. Enrichment Analysis of GO Terms for the DEGs

**Figure 3:**
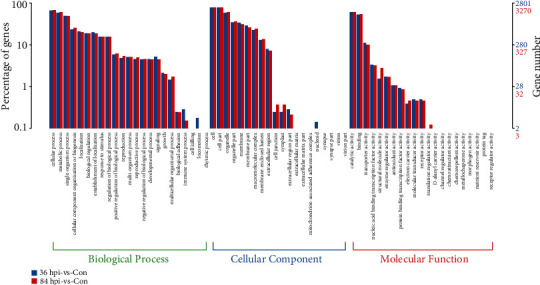
Functional annotation of the DEGs based on GO categorization. Blue columns represent the number of DEGs in 36 hpi-vs-Con. Red columns represent the number of DEGs in 84 hpi-vs-Con. The *x*-axis has descriptions of the corresponding GO terms.

The DEGs were subjected to a GO analysis to understand their functional differences. A corrected *p* value below 0.05 indicated that the function was enriched. Using Blast2GO software, the GO terms were organized into three ontologies: biological process, cellular component, and molecular function (Figure 3). The GO enrichment classification revealed that biological processes were dominated by cellular, metabolic, and single-organism processes. For the cellular component category, the DEGs assigned to the cell, cell part, and organelle were the most abundant. In the molecular function category, the DEGs related to catalytic activity and binding were the most abundant in both phases. The number of upregulated genes involved in structural molecule activity significantly increased in 84 hpi-vs-Con (76 DEGs) compared to 36 hpi-vs-Con (22 DEGs), suggesting the importance of the functions associated with these genes in the necrotrophic phase.

#### 3.4.2. KEGG Metabolic Pathway Enrichment Analysis of DEGs

**Figure 4:**
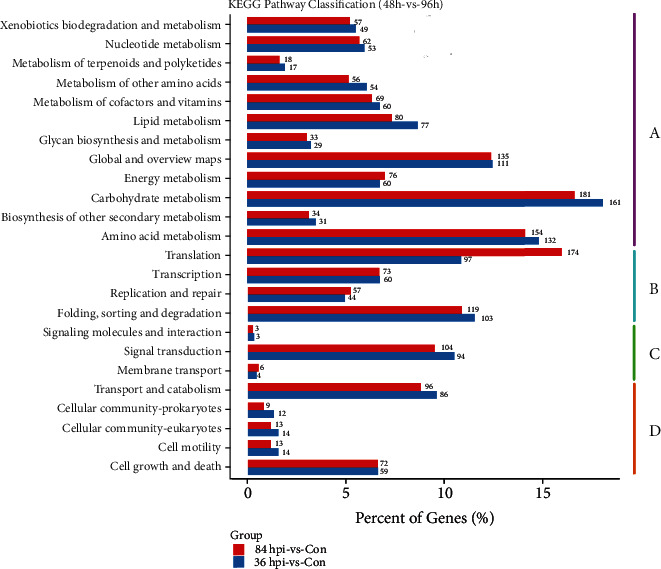
KEGG enrichment analysis of DEGs. The DEGs were clustered into four categories. (a) Metabolism. (b) Genetic information processing. (c) Environmental information processing. (d) Cellular processes. The *y*-axis has descriptions of the corresponding KEGG terms.

To elucidate the significantly enriched biochemical pathways of DEGs in *S*. *lycopersici*, an enrichment analysis by comparing both 36 hpi and 84 hpi with Con was performed using the KEGG database. The DEGs in 36 hpi-vs-Con and 84 hpi-vs-Con clustered into four categories, with the most represented classification being metabolic categories (Figure 4). Carbohydrate metabolism (161 and 181 DEGs, respectively), amino acid metabolism (132 and 154 DEGs, respectively), and lipid metabolism (111 and 135 DEGs, respectively) were the main metabolic pathways. In the genetic information processing categories, the most significantly enriched KEGG pathways in 36 hpi-vs-Con and 84 hpi-vs-Con were translation (97 and 174 DEGs, respectively) and folding, sorting, and degradation (103 and 119 DEGs, respectively). The KEGG cellular processing categories included two main pathways in 36 hpi-vs-Con and 84 hpi-vs-Con, including transport and catabolism (86 and 96 DEGs, respectively) and cell growth and death (59 and 72 DEGs, respectively). Additionally, the most abundant subcategory in KEGG environmental information processing was signal transduction (94 and 104 DEGs, respectively).

**Figure 5:**
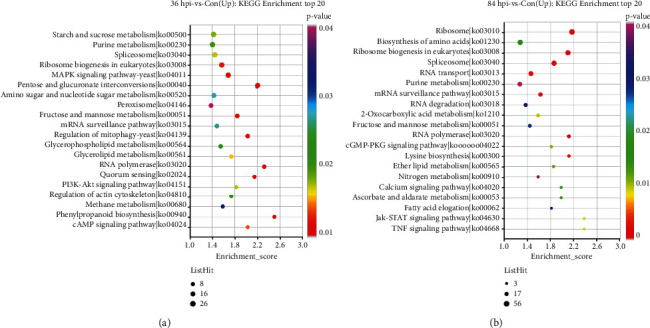
KEGG enrichment analysis of the upregulated DEGs of *S*. *lycopersici*. (a) The top 20 enriched KEGG terms of the upregulated genes in 36 hpi-vs-Con. (b) The top 20 enriched KEGG terms of the upregulated genes in 84 hpi-vs-Con. The *y*-axis has descriptions of the corresponding KEGG terms.

The upregulated DEGs in 36 hpi-vs-Con and 84 hpi-vs-Con were significantly enriched in 44 and 24 pathways, respectively (*p* value < 0.05). The KEGG enrichment analysis revealed that the greatest numbers of upregulated genes were involved in the starch and sucrose metabolic pathway (ko: 00500) and purine metabolic pathway (ko: 00230), with 26 upregulated DEGs enriched in both pathways in 36 hpi-vs-Con, followed by the spliceosome (ko: 03040, 25 upregulated DEGs), ribosome biogenesis in eukaryotes (ko: 03008, 24 upregulated DEGs), mitogen-activated protein kinase (MAPK) signaling pathway (ko: 04011, 22 upregulated DEGs), and pentose and glucuronate interconversion pathway (ko: 00040, 19 upregulated DEGs) (Figure 5). Compared with the 36 hpi-vs-Con group, there were some changes in the enriched pathways in 84 hpi-vs-Con. The six pathways having the most DEGs (at no less than 30) were the ribosome (ko: 03010, 56 upregulated DEGs), biosynthesis of amino acids (ko: 01230, 43 upregulated DEGs), ribosome biogenesis in eukaryotes (ko: 03008, 41 upregulated DEGs), spliceosome (ko: 03040, upregulated DEGs), RNA transport (ko: 03013, 35 upregulated DEGs), and purine metabolism (ko: 00230, 30 upregulated DEGs).

### 3.5. RNA-seq Validation by qRT-PCR

**Figure 6:**
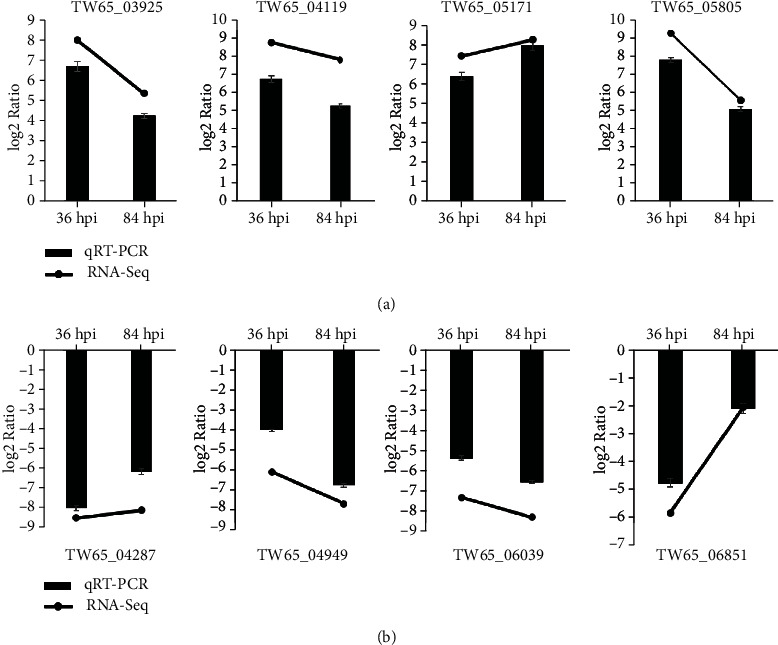
Validation of the expression levels of DEGs in *S*. *lycopersici* by qRT-PCR. Relative expression of 4 genes was upregulated (a), while the other 4 genes were downregulated (b) at 36 hpi and 84 hpi.

To validate the RNA-seq data, eight randomly selected genes were examined to confirm their expression dynamics using qRT-PCR. The qRT-PCR results revealed expression patterns for all eight genes that were consistent with the RNA-seq data (Figure 6), which confirmed the change trends in expression detected in the RNA-seq analyses. These results showed a high correlation between the RNA-seq and qRT-PCR results, indicating that the RNA-seq data were reliable.

### 3.6. Genes Associated with Pathogenesis

#### 3.6.1. Genes Associated with Cell Wall-Degrading Enzymes (CWDEs)

**Figure 7:**
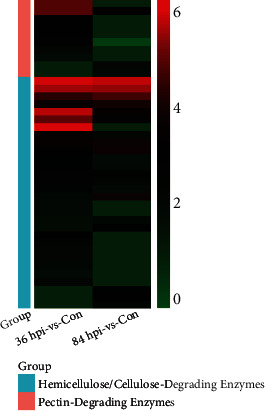
A heatmap of differentially expressed genes associated with CWDEs. Each small square represents a CWDE-associated DEG, and the color represents the log2 fold change of gene differential expression. Red indicates genes with high expression levels in 36/84 hpi-vs-Con. Green indicates genes with low expression levels in 36/84 hpi-vs-Con.

In plant pathogenic fungi, CWDEs have important roles in penetrating and colonizing their hosts. In this study, a number of CWDE-encoding genes displayed differential expression during the early infection (36 hpi-vs-Con group) and necrotrophic (84 hpi-vs-Con group, Table S1) phases. Nonsupervised clustering analyses were performed, and a gene expression heatmap was constructed to obtain a more precise overview (Figure 7). In tomato leaves, eight genes associated with pectin-degrading enzymes were induced during the early infection phase, and seven showed nondifferential expression or were downregulated during the necrotrophic phase. The top two upregulated genes were TW65_06206 encoding pectate lyase and TW65_09220 encoding pectinesterase with log2 fold changes of 5.11 and 5.08 (Table S1), respectively. In total, 17 cellulose-degrading enzyme-coding genes were upregulated during the early infection phase, and 3 of these genes simultaneously function to degrade hemicellulose and cellulose. Among the 17 upregulated genes during the necrotrophic phase, nine genes showed higher expression levels than during the early infection phase. There were 13 upregulated genes during the early infection phase that functioned to degrade hemicellulose, but almost all of them showed relatively lower expression levels during the necrotrophic phase, except for TW65_07268.

On the whole, a number of *S*. *lycopersici* genes associated with CWDEs had diverse expression patterns during the pathogenic process on tomato leaves. Their combined expression may help impair plant cell structures and provide nutrients for pathogen growth. Pectin-degrading enzyme-related genes were upregulated mainly during the early stages of infection. The upregulated expression levels of hemicellulose- and cellulose-degrading enzyme-coding genes were maintained for relatively long times. In addition, different cellulose-degrading enzyme-coding genes were induced during different interaction periods.

#### 3.6.2. Genes Involved in Adhesion and Colonization

**Figure 8:**
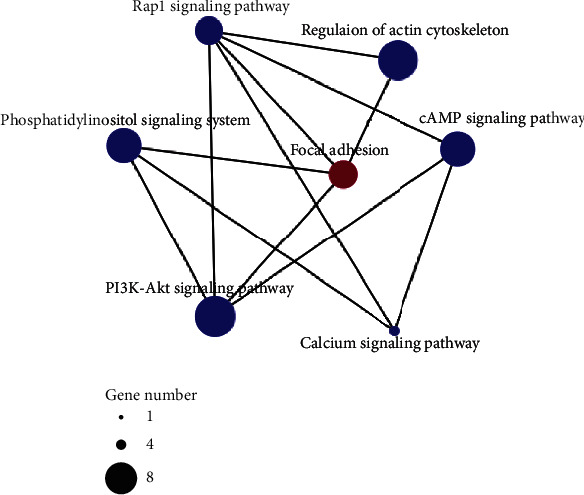
Network map of metabolic pathways associated with the focal adhesion pathway. The KEGG-based metabolic pathway network map of enriched upregulated genes in 36 hpi-vs-Con. The node sizes represent the numbers of enriched upregulated genes.

Genes related to adhesion and colonization also play important roles in pathogenesis [31]. The KEGG analysis showed that seven upregulated genes (TW65_01775, TW65_02246, TW65_04589, TW65_07048, TW65_07418, TW65_08271, and TW65_08475) were significantly enriched in the focal adhesion pathway (ko: 04510) in 36 hpi-vs-Con (*p* value < 0.05). The network map of the metabolic pathways suggested that in 36 hpi-vs-Con, six important pathways significantly enriched in the KEGG analysis were strongly associated with the focal adhesion pathway (*p* value < 0.05). These six pathways included the actin cytoskeleton pathway (ko: 04810) and five signaling pathways, PI3K-Akt (ko: 04151), cyclic adenosine monophosphate (cAMP) (ko: 04024), phosphatidylinositol (ko: 04070), Rap1 (ko: 04015), and calcium (ko: 04020) (Figure 8 and Table S2). Our study showed that six of these seven closely related pathways were uniquely enriched in 36 hpi-vs-Con, indicating that the expression of related genes was associated with pathogen-host interactions during the early infection phase.

#### 3.6.3. Genes Involved in Signal Regulation

Specific sensors/receptors and intricate coordination in cell signaling are considered to play major roles in pathogenic fungi associated with cell recognition and initial invasive structure formation when invading and infecting their plant hosts. In our study, one Pth11-like integral membrane protein (TW65_02983), a putative sensor/receptor, was found to have upregulated expression in 36 hpi-vs-Con, but no significant increase was seen in 84 hpi-vs-Con, indicating that it was required mainly during the early periods of the pathogenic process for cell identification (Table S3).

Protein kinases play a major role in cell signals (including MAPK, cAMP, and calcium), which function in some major signaling pathways and are associated with virulence in phytopathogenic fungi, such as affecting adhesion [31]. The transcriptomic analysis suggested that the expression levels of several protein kinases, such as serine/threonine protein kinase (TW65_00823, TW65_01420, TW65_01775, TW65_05069, TW65_07479, and TW65_98149), histidine kinase (TW65_01772, TW65_02044, and TW65_04432), phosphatidylinositol 3-kinase (TW65_06373), and inositol monophosphatase (TW65_08114), increased during the pathogenic process (Table S3).

MAPK cascades play pivotal roles in the infection-associated development of fungi [32]. Two genes (TW65_06425 and TW65_71836) encoding mitogen-activated protein kinases, which are vital in MAPK cascades, also showed higher expression levels during the pathogenic phase. Thus, these signal regulation-associated genes may play important roles during the pathogenic process.

#### 3.6.4. Genes Associated with Fungal Proteases

Proteases are important for phytopathogenic fungi during different aspects of the infection process, such as adhesion, initial penetration, and colonization [33]. In this study, the upregulation of serine proteases (TW65_01279 and TW65_04285) and metalloproteinases (TW65_02892 and TW65_08011) during the pathogenic phase may be beneficial to adhesion and pathogenicity (Table S4).

## 4. Discussion

*S*. *lycopersici* is a necrotrophic phytopathogenic fungus capable of infecting a wide range of plants and causing leaf spot disease [34], which has transitioned from minor to major disease, especially on facility-grown tomatoes over the last few years. Therefore, the pathogenic mechanisms of *S*. *lycopersici* against plants should be studied. Here, we reported the infection-related transcriptome of a *S*. *lycopersici* isolate sampled from tomato. Various candidate pathogenicity determinants of *S*. *lycopersici* were found during its pathogenesis on tomato. The expression dynamics of these genes at different periods illustrated their important roles in infection, colonization, pathogenicity, and survival in a hostile plant environment. These results may facilitate the study of the pathogenic mechanism of *S*. *lycopersici* in tomato.

Plant tissue penetration is a prerequisite for plant pathogenic fungi to infect and colonize a potential host [35], and CWDEs are particularly important, especially for necrotrophs and fungi that have no specialized penetration structures [36]. In addition, CWDEs are required for all phytopathogenic fungi during the late stages of host invasion [37]. In this study, a number of genes encoding CWDEs were induced during the pathogenesis of *S*. *lycopersici*. In total, 80% of upregulated pectin-degrading enzyme-associated genes were induced during the early infection phase. A total of 30 hemicellulose- and cellulose-degrading enzyme-related genes were induced during pathogenesis. Among them, 20 upregulated genes in each of the infection stages indicated their ubiquitous and indispensable roles during the progression of gray leaf spot disease in tomato. Pectinolytic enzymes are the first set of degradative enzymes secreted during infection, and they may assist in invasion and contribute to virulence in a wide range of pathogens [38]. Cellulases and hemicellulases may also contribute to virulence during infection. Van Vu et al. reported that the cellulases of *Magnaporthe oryzae* contribute to host penetration and further invasion [39]. Yang et al. showed that in *Botrytis cinerea*, the cell death activity of the xylanase BcXyl1 is independent of its xylanase activity, suggesting that BcXyl1 not only contributes to *B*. *cinerea* virulence but also induces plant defense responses [40]. Therefore, it is tempting to speculate that *S*. *lycopersici* is utilizing differential sets of CWDEs to promote pathogenesis by promoting cell wall damage under varying physiological conditions. The genes showing different expression dynamics may play different roles in pathogenesis. Here, pectin-degrading enzymes were necessary during the early stages of the infection, and hemicellulose- and cellulose-degrading enzymes were indispensable during the entire pathogenic period. It is worth noting that we found one gene encoding the polygalacturonase SlPG1 (TW65_07585), which was especially induced during the early infection phase. An *Aspergillus flavus* strain carrying a deletion of the polygalacturonase *pecA* gene reduces lesion development in cotton [41]. A single Bcpg1 is required for the full virulence of *B*. *cinerea* against different hosts [42]. However, the relevance of SlPG1 to the infection and pathogenesis of *S*. *lycopersici* requires further investigation.

The transcriptome analysis in this study revealed that a number of adhesion pathway-related genes closely correlated with the actin cytoskeleton and signal transduction were mainly induced during the early infection phase. The KEGG analysis showed that seven upregulated genes during the early infection phase were significantly enriched in the focal adhesion pathway (ko: 04510). Additionally, the KEGG analysis revealed that during the early infection phase, there were six important significantly enriched pathways strongly associated with the focal adhesion pathway. These included five important signaling pathways. Furthermore, two genes encoding an activating transcription factor (*TW65_00541*) and a transcription factor (*TW65_06260*), respectively, were significantly induced. The function of adhesion is thought to be essential for a fungal pathogen's normal prepenetration development and for successful infection [43]. First, firm attachments enable spores to avoid being washed from the host plant's surface. Then, the proper recognition of the topographic signals is necessary for properly oriented germ tube growth and appressorial differentiation [43]. Furthermore, adhesion continues to be important for intercellular development, cell wall penetration, and host tissue colonization as the hyphae enter the substomatal chambers [43]. The adhesion-deficient mutants of *Fusarium solani* f. sp. *cucurbitae* have greatly reduced virulence levels on nonwounded zucchini fruit, confirming the importance of adhesion as a virulence factor [44]. In *Saccharomyces cerevisiae*, the *FLO1* and *FLO11* genes responsible for adhesion and initial surface adhesion, respectively, are activated by the transcription factor Flo8 under the control of the cAMP/PKA signal transduction pathway [45, 46], which controls adhesion, colonization, and pseudohyphal development [45, 47]. Thus, we speculated that genes involved in the focal adhesion pathway of *S*. *lycopersici* are conducive to spore adhesion, bud tube directional growth, appressorial formation, and cell wall contact in the substomatic cavity during the interaction establishment phase. In addition, many important signaling pathways are involved in regulating the expression of adhesion-related genes.

During the establishment of an interaction between the pathogen and the host plant, receptors and sensors are utilized to sense and respond to the physicochemical cues of the host surface, which trigger the initiation of pathogenic development in fungi. Several intracellular signaling pathways, such as G-protein and cAMP/PKA, as well as MAPK cascades that function downstream, regulate the formation of infection-related structures required for host penetration [32]. One gene for a predicted receptor/sensor PTH11-like integral membrane protein (TW65_02983) was found to have upregulated expression exclusively during the early infection phase. *PTH11* was identified in *M*. *oryzae*, and it may be required for completing appressorial morphogenesis. A significant proportion (>85%) of the *pth11△* mutant failed in appressorial formation [48]. Furthermore, Pth11 is essential for pathogenesis as a bona fide G-protein-coupled receptor that functions upstream of the cAMP-dependent signaling pathway. Several histidine kinases and other protein kinases were upregulated or differentially expressed in our study. Biochemical studies have revealed that histidine kinases in phytopathogenic bacteria sense not only environmental stimuli but also important host plant chemicals, suggesting the existence of mutual communication between phytopathogenic plants and bacteria [49]. The overexpression of the hybrid histidine kinase DRK1 in the yeast phase of *Sporothrix schenckii* is involved in the regulation of the mycelium-to-yeast transition and required for pathogenesis [50]. Additionally, we found two MAPK-encoding genes showing high expression levels during the pathogenic phase. MAPK cascades play pivotal roles in appressorial formation and pathogenicity in phytopathogenic fungi. MAPK cascades comprise three kinds of conserved kinases, MAP kinase kinase kinase (MAPKKK), MAP kinase kinase (MAPKK), and MAPK, which function downstream of G-protein signaling and cAMP/PKA pathways [33]. In *M*. *oryzae*, the MAPKKK Mst11, the MAPKK Mst7, the MAPK Pmk1, and the adaptor protein Mst50 function as an Mst11-Mst7-Pmk1 cascade to regulate appressorial development [51]. In *B*. *cinerea*, the deletion of *BMP1*, *Ste7*, *Ste11*, or *Ste50* causes defects in the development of infection-related structures, confirming that BMP1 MAPK signaling is likely regulated by the surface sensor Msb2 [52–54]. Similarly, He et al. and Wang et al. demonstrated that the MAPK CgMK1 and its upstream components MAPKKK CgSte11 and MAPKK CgSte7, as well as the putative adaptor protein CgSte50, play critical roles in appressorial formation, invasive growth, cellophane membrane penetration, and pathogenicity of the hemibiotrophic pathogen *Colletotrichum gloeosporioides* [55, 56]. Thus, *S*. *lycopersici* is likely to perceive and interact with host cells through receptors/sensors, histidine kinases, and other protein kinases. Then, MAPK cascades function downstream of G-protein signaling and cAMP/PKA pathways, thereby regulating appressorial formation and pathogenicity on tomato.

A few fungal protease-encoding genes of *S*. *lycopersici* were identified as being induced during pathogenesis on tomato in our study. The correlation between proteolytic activity and pathogenicity has been shown for several phytopathogenic fungi in the establishment of disease [57, 58]. The subtilisin-like serine proteases FgPrb1 of *Fusarium graminearum* and Aaprb1 of *Alternaria alternata* are required for fungal pathogenesis [59, 60]. In addition, several fungal secreted proteases and metalloproteases possess functions that cleave specific plant chitinases. For example, both the secreted metalloprotease FoMep1 and the serine protease FoSep1 discovered from tomato pathogen *F*. *oxysporum* f. sp. *lycopersici* are responsible for reducing the chitinase activity in tomato [61]. This suggests that during pathogenesis, *S*. *lycopersici* may upregulate its arsenals to protect its hyphae from degradation by the host defensive cell wall-degrading enzymes.

## 5. Conclusions

In conclusion, here, the RNA-seq of *S*. *lycopersici* from tomato was presented, and a large number of candidate pathogenicity determinants of *S*. *lycopersici* were found that may play crucial roles during the establishment of tomato gray leaf spot disease. However, the functions of these genes need to be confirmed using homologous recombination, insertional mutagenesis, and RNAi-based gene silencing. Our results not only facilitate an understanding of the pathogenic molecular mechanisms of *S*. *lycopersici* in tomato but also lay a foundation for disease control.

## Figures and Tables

**Table 1:** Summary of the RNA-seq data.

cDNA library	Raw reads (M)	Clean reads (M)	Raw bases (Gb)	Clean bases (Gb)	Q30^1^ (%)
Con (mean)	48.23	45.74	7.23	6.55	92.68
36 hpi (mean)	49.60	46.93	7.44	6.72	92.52
84 hpi (mean)	49.18	47.09	7.38	6.76	93.09

Note: ^1^Q30: percentage of bases with a Phred value > 30. (mean) indicates the mean of three biological replicates.

## Data Availability

The RNA-seq data has been deposited in China National GeneBank (CNGB, https://www.cngb.org/index.html) under project number CNP0002533.

## Supplementary Materials

Supplementary Materials Table S1: the upregulated genes involved in CWDEs. Table S2: the KEGG analysis of the upregulated genes enriched in metabolic pathways associated with the focal adhesion pathway in 36 hpi-vs-Con. Table S3: the upregulated genes involved in signal reception and regulation. Table S4: the upregulated genes associated with fungal proteases.

## References

[1] Simmons E. G. (2001). Perfect states of Stemphylium—IV. *Harvard Papers in Botany*.

[2] Kim B.-S., Cho H.-J., Hwang H.-S., Cha Y.-S. (1999). Gray leaf spot of tomato caused by Stephylium solani. *The Plant Pathology Journal*.

[3] Yang H., Zhao T., Jiang J. (2017). Mapping and screening of the tomato Stemphylium lycopersici resistance gene, Sm, based on bulked segregant analysis in combination with genome resequencing. *BMC Plant Biology*.

[4] Su X., Zhu G., Huang Z. (2019). Fine mapping and molecular marker development of the Sm gene conferring resistance to gray leaf spot (Stemphylium spp.) in tomato. *Theoretical and Applied Genetics*.

[5] Weber G. F. (1930). Gray leaf spot of tomato caused by Stemphylium solani, sp. nov. *Phytopathology*.

[6] de Miranda B. E. C., Boiteux L. S., Reis A. (2010). Identification of Solanum (section Lycopersicon) accessions with resistance to Stemphylium solani and S. lycopersici. *Horticultura Brasileira*.

[7] Franco M. E. E., Troncozo M. I., López S. M. Y. (2017). A survey on tomato leaf grey spot in the two main production areas of Argentina led to the isolation of Stemphylium lycopersici representatives which were genetically diverse and differed in their virulence. *European Journal of Plant Pathology*.

[8] Nasehi A., Al-Sadi A. M., Esfahani M. N. (2019). Molecular re-identification of Stemphylium lycopersici and Stemphylium solani isolates deposited in NCBI GenBank and morphological characteristics of Malaysian isolates. *European Journal of Plant Pathology*.

[9] Xie X., Wu J., Cheng Y. (2019). First report of Stemphylium lycopersici causing leaf spot on hot pepper in China. *Canadian Journal of Plant Pathology*.

[10] Liu H., Wang H., Zhong J. (2019). First report of Stemphylium lycopersici and Stemphylium vesicarium causing leaf spot on lettuce (Lactuca sativa) in China. *Plant Disease*.

[11] Yang H., Li Y., He Y. (2020). First report of Stemphylium lycopersici causing gray leaf spot on physali (Physalis alkekengi) in China. *Plant Disease*.

[12] Tomioka K., Abe D., Kawaguchi A. (2021). Stemphylium leaf spot of asparagus caused by Stemphylium lycopersici. *Journal of General Plant Pathology*.

[13] Ben H., Huo J., Yao Y. (2021). Stemphylium lycopersici causing leaf spot of watermelon (Citrullus lanatus) in China. *Plant Disease*.

[14] Kim B.-S., Yu S.-H., Cho H.-J., Hwang H.-S. (2004). Gray leaf spot in peppers caused by Stemphylium solani and S. lycopersici. *Plant Pathology Journal*.

[15] Bentes J. L., Matsuoka K. (2005). Histology of Stemphylium solani and tomato interaction. *Fitopatologia Brasileira*.

[16] Nasehi A., Kadir J.-B., Nasr-Esfahani M. (2014). Analysis of genetic and virulence variability of Stemphylium lycopersici associated with leaf spot of vegetable crops. *European Journal of Plant Pathology*.

[17] Medina R., Franco M. E., da Cruz Cabral L. (2021). The secondary metabolites profile of Stemphylium lycopersici, the causal agent of tomato grey leaf spot, is complex and includes host and non-host specific toxins. *Australasian Plant Pathology*.

[18] Franco M. E., López S., Medina R., Saparrat M. C., Balatti P. (2015). Draft genome sequence and gene annotation of Stemphylium lycopersici strain CIDEFI-216. *Genome Announcements*.

[19] Medina R., Franco M. E. E., Lucentini G., Saparrat M. C. N., Balatti P. A. (2018). Draft genome sequences of sporulating (CIDEFI-213) and nonsporulating (CIDEFI-212) strains of Stemphylium lycopersici. *Microbiology resource announcements*.

[20] Zeng R., Gao S., Xu L., Liu X., Dai F. (2018). Prediction of pathogenesis-related secreted proteins from Stemphylium lycopersici. *BMC Microbiology*.

[21] Wang Z., Gerstein M., Snyder M. (2009). RNA-Seq: a revolutionary tool for transcriptomics. *Nature Reviews Genetics*.

[22] Cho H., Kim B., Hwang H. (2001). Resistance to gray leaf spot in Capsicum peppers. *HortScience*.

[23] Bolger A. M., Lohse M., Usadel B. (2014). Trimmomatic: a flexible trimmer for Illumina sequence data. *Bioinformatics*.

[24] Grabherr M. G., Haas B. J., Yassour M. (2011). Full-length transcriptome assembly from RNA-Seq data without a reference genome. *Nature Biotechnology*.

[25] Pertea G., Huang X., Liang F. (2003). TIGR Gene Indices clustering tools (TGICL): a software system for fast clustering of large EST datasets. *Bioinformatics*.

[26] Conesa A., Götz S., García-Gómez J. M., Terol J., Talón M., Robles M. (2005). Blast2GO: a universal tool for annotation, visualization and analysis in functional genomics research. *Bioinformatics*.

[27] Li R., Yu C., Li Y. (2009). SOAP2: an improved ultrafast tool for short read alignment. *Bioinformatics*.

[28] Love M. I., Huber W., Anders S. (2014). Moderated estimation of fold change and dispersion for RNA-seq data with DESeq2. *Genome Biology*.

[29] Boyle E. I., Weng S., Gollub J. (2004). GO::TermFinder--open source software for accessing gene ontology information and finding significantly enriched gene ontology terms associated with a list of genes. *Bioinformatics*.

[30] Pfaffl M. W. (2001). A new mathematical model for relative quantification in real-time RT–PCR. *Nucleic Acids Research*.

[31] He D., Zhang X., Gao S., You H., Zhao Y., Wang L. (2021). Transcriptome analysis of dimorphic fungus Sporothrix schenckii exposed to temperature stress. *International Microbiology*.

[32] Kou Y., Naqvi N. I. (2016). Surface sensing and signaling networks in plant pathogenic fungi. *Seminars in Cell & Developmental Biology*.

[33] Soberanes-Gutiérrez C. V., Juárez-Montiel M., Olguín-Rodríguez O., Hernández-Rodríguez C., Ruiz-Herrera J., Villa-Tanaca L. (2015). The pep4 gene encoding proteinase A is involved in dimorphism and pathogenesis of Ustilago maydis. *Molecular Plant Pathology*.

[34] Medina R., Franco M. E., Lucentini C. G. (2019). Secondary metabolites synthesized by *Stemphylium lycopersici* and *Fulvia fulva*, necrotrophic and biotrophic fungi pathogen of tomato plants. *Current Plant Biology*.

[35] Bandara Y., Weerasooriya D., Liu S., Little C. (2018). The necrotrophic fungus Macrophomina phaseolina promotes charcoal rot susceptibility in grain sorghum through induced host cell-wall-degrading enzymes. *Phytopathology*.

[36] Łaźniewska J., Macioszek V. K., Kononowicz A. K. (2012). Plant-fungus interface: the role of surface structures in plant resistance and susceptibility to pathogenic fungi. *Physiological and Molecular Plant Pathology*.

[37] Gibson D. M., King B. C., Hayes M. L., Bergstrom G. C. (2011). Plant pathogens as a source of diverse enzymes for lignocellulose digestion. *Current Opinion in Microbiology*.

[38] Zhang L., van Kan J. A. (2013). Pectin as a barrier and nutrient source for fungal plant pathogens. *Agricultural Applications*.

[39] Van Vu B., Itoh K., Nguyen Q. B., Tosa Y., Nakayashiki H. (2012). Cellulases belonging to glycoside hydrolase families 6 and 7 contribute to the virulence of Magnaporthe oryzae. *Molecular Plant-Microbe Interactions*.

[40] Yang Y., Yang X., Dong Y., Qiu D. (2018). The Botrytis cinerea xylanase BcXyl1 modulates plant immunity. *Frontiers in Microbiology*.

[41] Shieh M.-T., Brown R. L., Whitehead M. P. (1997). Molecular genetic evidence for the involvement of a specific polygalacturonase, P2c, in the invasion and spread of Aspergillus flavus in cotton bolls. *Applied and Environmental Microbiology*.

[42] Have A. T., Mulder W., Visser J., van Kan J. A. (1998). The endopolygalacturonase gene Bcpg1 is required for full virulence of Botrytis cinerea. *Molecular Plant-Microbe Interactions*.

[43] Braun E., Howard R. (1994). Adhesion of fungal spores and germlings to host plant surfaces. *Protoplasma*.

[44] Jones M. J., Epstein L. (1990). Adhesion of macroconidia to the plant surface and virulence of Nectria haematococca. *Applied and Environmental Microbiology*.

[45] Rupp S., Summers E., Lo H. J., Madhani H., Fink G. (1999). MAP kinase and cAMP filamentation signaling pathways converge on the unusually large promoter of the yeast FLO11 gene. *The EMBO Journal*.

[46] Fichtner L., Schulze F., Braus G. H. (2007). Differential Flo8p-dependent regulation of FLO1 and FLO11 for cell–cell and cell–substrate adherence of S. cerevisiae S288c. *Molecular Microbiology*.

[47] Lin C.-J., Sasse C., Gerke J. (2015). Transcription factor SomA is required for adhesion, development and virulence of the human pathogen Aspergillus fumigatus. *PLoS Pathogens*.

[48] DeZwaan T. M., Carroll A. M., Valent B., Sweigard J. A. (1999). Magnaporthe grisea pth11p is a novel plasma membrane protein that mediates appressorium differentiation in response to inductive substrate cues. *The Plant Cell*.

[49] Wang F.-F., Qian W. (2019). The roles of histidine kinases in sensing host plant and cell–cell communication signal in a phytopathogenic bacterium. *Philosophical Transactions of the Royal Society B*.

[50] Zhang Z., Hou B., Wu Y. Z., Wang Y., Liu X., Han S. (2018). Two-component histidine kinase DRK1 is required for pathogenesis in Sporothrix schenckii. *Molecular Medicine Reports*.

[51] Li G., Zhang X., Tian H., Choi Y. E., Tao W. A., Xu J. R. (2017). Mst50 is involved in multiple MAP kinase signaling pathways in Magnaporthe oryzae. *Environmental Microbiology*.

[52] Zheng L., Campbell M., Murphy J., Lam S., Xu J.-R. (2000). The BMP1 gene is essential for pathogenicity in the gray mold fungus Botrytis cinerea. *Molecular Plant-Microbe Interactions*.

[53] Schamber A., Leroch M., Diwo J., Mendgen K., Hahn M. (2010). The role of mitogen-activated protein (MAP) kinase signalling components and the Ste12 transcription factor in germination and pathogenicity of Botrytis cinerea. *Molecular Plant Pathology*.

[54] Leroch M., Mueller N., Hinsenkamp I., Hahn M. (2015). The signalling mucin Msb2 regulates surface sensing and host penetration via BMP1 MAP kinase signalling in Botrytis cinerea. *Molecular Plant Pathology*.

[55] He P., Wang Y., Wang X., Zhang X., Tian C. (2017). The mitogen-activated protein kinase CgMK1 governs appressorium formation, melanin synthesis, and plant infection of Colletotrichum gloeosporioides. *Frontiers in Microbiology*.

[56] Wang X., Lu D., Tian C. (2021). Mitogen-activated protein kinase cascade CgSte50-Ste11-Ste7-Mk1 regulates infection-related morphogenesis in the poplar anthracnose fungus *Colletotrichum gloeosporioides*. *Microbiological Research*.

[57] Bindschedler L. V., Sanchez P., Dunn S. (2003). Deletion of the *SNP1* trypsin protease from *Stagonospora nodorum* reveals another major protease expressed during infection. *Fungal Genetics and Biology*.

[58] Olivieri F., Maldonado S., Tonon C., Casalongue C. (2004). Hydrolytic activities of Fusarium solani and Fusarium solani f. sp. eumartii associated with the infection process of potato tubers. *Journal of Phytopathology*.

[59] Xu L., Wang H., Zhang C. (2020). System-wide characterization of subtilases reveals that subtilisin-like protease FgPrb1 of *Fusarium graminearum* regulates fungal development and virulence. *Fungal Genetics and Biology*.

[60] Fu H., Chung K.-R., Liu X., Li H. (2020). *Aaprb1* , a subtilsin-like protease, required for autophagy and virulence of the tangerine pathotype of *Alternaria alternata*. *Microbiological Research*.

[61] Jashni M. K., Dols I. H., Iida Y. (2015). Synergistic action of a metalloprotease and a serine protease from Fusarium oxysporum f. sp. lycopersici cleaves chitin-binding tomato chitinases, reduces their antifungal activity, and enhances fungal virulence. *Molecular Plant-Microbe Interactions*.

