# Supplementary material for: Pathogenic Process-Associated Transcriptome Analysis of Stemphylium lycopersici from Tomato
Source: Int J Genomics. 2022 May 20;2022:4522132. doi: 10.1155/2022/4522132 (PMC9142275; doi:10.1155/2022/4522132)
Supplement: Supplementary Materials — Table S1: the upregulated genes involved in CWDEs. Table S2: the KEGG analysis of the upregulated genes enriched in metabolic pathways associated with the focal adhesion pathway in 36 hpi-vs-Con. Table S3: the upregulated genes involved in signal reception and regulation. Table S4: the upregulated genes associated with fungal proteases. [file 4522132.f1.zip › 4522132.f1/Table S1.docx]

Table S1: The up-regulated genes involved in CWDEs.

| Gene ID | log2FoldChange | | CWDE-related activity |
| --- | --- | --- | --- |
|  | 36 hpi | 84 hpi |  |
| TW65_06206 | 5.11 | - | Pectin-Degrading Enzymes |
| TW65_09220 | 5.08 | 3.03 | Pectin-Degrading Enzymes |
| TW65_05277 | 2.89 | - | Pectin-Degrading Enzymes |
| TW65_07585 | 2.77 | - | Pectin-Degrading Enzymes |
| TW65_01797 | 2.62 | - | Pectin-Degrading Enzymes |
| TW65_05785 | 2.49 | -1.22 | Pectin-Degrading Enzymes |
| TW65_01682 | 1.67 | - | Pectin-Degrading Enzymes |
| TW65_06566 | 1.22 | - | Pectin-Degrading Enzymes |
| TW65_02084 | - | 1.56 | Pectin-Degrading Enzymes |
| TW65_04718 | - | 1.38 | Pectin-Degrading Enzymes |
| TW65_01891 | 6.36 | 6.15 | Hemicellulose-Degrading Enzymes |
| TW65_07268 | 4.44 | 4.81 | Hemicellulose-Degrading Enzymes |
| TW65_92928 | 6.7 | - | Hemicellulose-Degrading Enzymes |
| TW65_08790 | 2.45 | 2.1 | Hemicellulose-Degrading Enzymes |
| TW65_04512 | 2.45 | 1.73 | Hemicellulose-Degrading Enzymes |
| TW65_03437 | 1.97 | 1.25 | Hemicellulose-Degrading Enzymes |
| TW65_08568 | 1.3 | 0.84 | Hemicellulose-Degrading Enzymes |
| TW65_05159 | 2.56 | - | Hemicellulose-Degrading Enzymes |
| TW65_05302 | 1.63 | - | Hemicellulose-Degrading Enzymes |
| TW65_06237 | 1.43 | - | Hemicellulose-Degrading Enzymes |
| TW65_02961 | 5.9 | 5.8 | Hemicellulose/Cellulose-Degrading Enzymes |
| TW65_01857 | 1.83 | 0.78 | Hemicellulose/Cellulose-Degrading Enzymes |
| TW65_03932 | 1.64 | -0.86 | Hemicellulose/Cellulose-Degrading Enzymes |
| TW65_04814 | 3.55 | 3.96 | Cellulose-Degrading Enzymes |
| TW65_06015 | 6.07 | 3.42 | Cellulose-Degrading Enzymes |
| TW65_05035 | 5.21 | 2.33 | Cellulose-Degrading Enzymes |
| TW65_06178 | 3.46 | 1.7 | Cellulose-Degrading Enzymes |
| TW65_03289 | 3.37 | 3.62 | Cellulose-Degrading Enzymes |
| TW65_04060 | 2.97 | 3.72 | Cellulose-Degrading Enzymes |
| TW65_00945 | 2.73 | 1.17 | Cellulose-Degrading Enzymes |
| TW65_08926 | 2.67 | 1.28 | Cellulose-Degrading Enzymes |
| TW65_00563 | 1.45 | 3.63 | Cellulose-Degrading Enzymes |
| TW65_09364 | 1.39 | 0.78 | Cellulose-Degrading Enzymes |
| TW65_06750 | 1.09 | 1.5 | Cellulose-Degrading Enzymes |
| TW65_98107 | 1.07 | 2.54 | Cellulose-Degrading Enzymes |
| TW65_02993 | 1.62 | - | Cellulose-Degrading Enzymes |
| TW65_02942 | 1.19 | - | Cellulose-Degrading Enzymes |
| TW65_01026 | - | 2.71 | Cellulose-Degrading Enzymes |
| TW65_00118 | - | 2.52 | Cellulose-Degrading Enzymes |
| TW65_06788 | - | 1.43 | Cellulose-Degrading Enzymes |

“-” indicates that the gene was not differentially expressed.
