# Supplementary material for: Pathogenic Process-Associated Transcriptome Analysis of Stemphylium lycopersici from Tomato
Source: Int J Genomics. 2022 May 20;2022:4522132. doi: 10.1155/2022/4522132 (PMC9142275; doi:10.1155/2022/4522132)
Supplement: Supplementary Materials — Table S1: the upregulated genes involved in CWDEs. Table S2: the KEGG analysis of the upregulated genes enriched in metabolic pathways associated with the focal adhesion pathway in 36 hpi-vs-Con. Table S3: the upregulated genes involved in signal reception and regulation. Table S4: the upregulated genes associated with fungal proteases. [file 4522132.f1.zip › 4522132.f1/Table S2.docx]

Table S2: The KEGG analysis of the up-regulated genes enriched in metabolic pathways associated with focal adhesion pathway in 36 hpi-vs-Con.

| KEGG ID | Term | ListHits | Up-regulated Gene ID |
| --- | --- | --- | --- |
| ko04510 | Focal adhesion | 7 | TW65_01775, TW65_02246, TW65_04589, TW65_07048, TW65_07418,  TW65_08271, TW65_08475 |
| ko04810 | Regulation of actin cytoskeleton | 9 | TW65_01775, TW65_01877, TW65_02246, TW65_05652, TW65_07048,  TW65_07418, TW65_07639, TW65_08271, TW65_08881 |
| ko04151 | PI3K-Akt signaling pathway | 9 | TW65_00540, TW65_01877, TW65_04589, TW65_05512, TW65_05791,  TW65_06373, TW65_06657, TW65_08204, TW65_08475 |
| ko04024 | cAMP signaling pathway | 8 | TW65_01103, TW65_01775, TW65_05116, TW65_06424, TW65_06554,  TW65_07048, TW65_07418, TW65_08271 |
| ko04070 | Phosphatidylinositol signaling system | 8 | TW65_00169, TW65_04112, TW65_04589, TW65_05116, TW65_05652,  TW65_07724, TW65_08114, TW65_08881 |
| ko04015 | Rap1 signaling pathway | 7 | TW65_01877, TW65_02246, TW65_04589, TW65_05116, TW65_06424,  TW65_06425, TW65_08271 |
| ko04020 | Calcium signaling pathway | 4 | TW65_04137, TW65_04589, TW65_05116, TW65_07724 |
