# Supplementary material for: Pathogenic Process-Associated Transcriptome Analysis of Stemphylium lycopersici from Tomato
Source: Int J Genomics. 2022 May 20;2022:4522132. doi: 10.1155/2022/4522132 (PMC9142275; doi:10.1155/2022/4522132)
Supplement: Supplementary Materials — Table S1: the upregulated genes involved in CWDEs. Table S2: the KEGG analysis of the upregulated genes enriched in metabolic pathways associated with the focal adhesion pathway in 36 hpi-vs-Con. Table S3: the upregulated genes involved in signal reception and regulation. Table S4: the upregulated genes associated with fungal proteases. [file 4522132.f1.zip › 4522132.f1/Table S3.docx]

Table S3: The up-regulated genes involved in signal reception and regulation.

| Gene ID | Description | FoldChange | |
| --- | --- | --- | --- |
|  |  | 36 hpi | 84 hpi |
| TW65_02983 | Pth11-like integral membrane protein | 2.85 | - |
| TW65_00823 | Serine threonine-protein kinase pak1 | 1.78 | 1.65 |
| TW65_01420 | Serine threonine-protein kinase nrc-2 | 5.92 | 3.11 |
| TW65_01775 | Serine threonine-protein kinase ste20 | 1.79 | 1.63 |
| TW65_05069 | Serine threonine-protein kinase rio1 | 1.87 | 5.59 |
| TW65_07479 | Serine threonine-protein kinase ssn3 | 1.76 | 1.87 |
| TW65_98149 | Serine threonine-protein kinase bur1 | 1.57 | 1.76 |
| TW65_01772 | Phytochrome-like histidine kinase phy1p | 1.76 | 2.46 |
| TW65_02044 | Two-component sensor protein histidine protein kinase | 1.57 | 1.97 |
| TW65_04432 | Two-component osmosensing histidine kinase | 2.40 | 2.69 |
| TW65_06373 | Phosphatidylinositol 3-kinase tor2 | 1.59 | 1.54 |
| TW65_08114 | Inositol monophosphatase protein | 2.35 | 5.25 |
| TW65_06425 | Mitogen-activated protein kinase | 4.55 | 4.80 |
| TW65_71836 | Mitogen-activated protein kinase mkc1 | 9.29 | 3.46 |

“-” indicates that the gene was not differentially expressed.
