# Supplementary material for: Pathogenic Process-Associated Transcriptome Analysis of Stemphylium lycopersici from Tomato
Source: Int J Genomics. 2022 May 20;2022:4522132. doi: 10.1155/2022/4522132 (PMC9142275; doi:10.1155/2022/4522132)
Supplement: Supplementary Materials — Table S1: the upregulated genes involved in CWDEs. Table S2: the KEGG analysis of the upregulated genes enriched in metabolic pathways associated with the focal adhesion pathway in 36 hpi-vs-Con. Table S3: the upregulated genes involved in signal reception and regulation. Table S4: the upregulated genes associated with fungal proteases. [file 4522132.f1.zip › 4522132.f1/Table S4.docx]

Table S4: The up-regulated genes associated with fungal proteases.

| Gene ID | Description | FoldChange | |
| --- | --- | --- | --- |
|  |  | 36 hpi | 84 hpi |
| TW65_01279 | Alkaline serine protease alp1 | 2.73 | 2.78 |
| TW65_04285 | Subtilisin-like serine protease | 7.93 | 2.21 |
| TW65_02892 | Zinc metalloproteinase | 4.53 | 3.09 |
| TW65_08011 | Zinc metalloproteinase | 3.10 | 4.81 |
